# Neopterin and biopterin levels and tryptophan degradation in patients with diabetes

**DOI:** 10.1038/s41598-020-74183-w

**Published:** 2020-10-12

**Authors:** Sinem Gürcü, Gözde Girgin, Göknur Yorulmaz, Bilge Kılıçarslan, Belgin Efe, Terken Baydar

**Affiliations:** 1 Department of Toxicology, Faculty of Pharmacy, Hacettepe University, 90-06230 Ankara, Turkey; 2 Eskisehir City Hospital, Hospital Pharmacy, Eskişehir, Turkey; 3 Faculty of Medicine, Department of Endocrinology, Osmangazi University, Eskişehir, Turkey

**Keywords:** Biological techniques, Immunology, Physiology, Endocrinology, Health occupations

## Abstract

This study aimed to evaluate the possible changes of neopterin, biopterin levels and tryptophan degradation in diabetes and to compare the results within diabetes groups and with healthy subjects. Diabetes mellitus patients and healthy controls were recruited the study. Patients were further subgrouped according to their drug therapy. Serum neopterin concentrations were detected by ELISA. Urinary neopterin, biopterin, serum tryptophan (Trp) and kynurenine (Kyn) levels were detected by HPLC. There was no difference between controls and diabetes patients in serum neopterin, urinary neopterin and biopterin levels (p > 0.05, all). Serum Trp and Kyn levels were significantly different in type 1 diabetes (T1DM) patients compared to controls (p < 0.05, both). Serum neopterin levels were significantly higher in type 2 diabetes patients (T2DM) compared to T1DM (p < 0.05). Urinary biopterin levels of T2DM patients using both metformin and vildagliptin were significantly higher than T1DM patients (p < 0.05). The correlations between serum neopterin and urinary neopterin, Kyn and Kyn/Trp were statistically significant in control and patient groups (p < 0.05, all). The study showed that Kyn/Trp was altered in diabetes patients due to immune modulation. On the other hand, although xenobiotic exposure may change pteridine levels, metformin and/or vildagliptin use in T2DM patients did not have any effect on the measured parameters.

## Introduction

Diabetes mellitus (diabetes), one of the biggest global health problems of the twenty-first century, is a chronic metabolic disease caused by absolute insulin deficiency or insulin effect disorder^1,2^. According to the 2015 data of the International Diabetes Federation Diabetes Atlas (IDF-DA), there are 318 million adults with impaired glucose tolerance at risk of developing diabetes in addition to 415 million adults with diabetes. By 2040, it is expected that one out of every 10 people would have diabetes^1^. The most common symptoms are polyuria, polydipsia, polyphagia or loss of appetite, weakness, dry mouth and nocturia. Retina, kidney, and peripheral nerves are commonly affected in diabetes patients. Atherosclerotic diseases of arteries are also increased and complications due to the altered blood supply of different compartments such as heart, brain, or lower extremities can be observed^3^. Diabetic cardiomyopathy is one of the major diabetic complications. There are 4 types of diabetes: Type 1 diabetes mellitus (T1DM) accounts for only 5–10% of those with diabetes and occurs frequently in children^1,2^. Type 2 diabetes mellitus (T2DM) starts with insulin resistance and progresses with insulin release disorder. The other two types of diabetes are gestational diabetes and diabetes caused by various pathologies and xenobiotics^1,4^. In T1DM, which is absolute insulin deficiency, only insulin preparations are used. Metformin is a biguanide derivative which is used in T2DM for more than 30 years^1,2,5^. Vildagliptin is an extremely selective, reversible dipeptidyl peptidase-4 (DDP_4_) inhibitor drug used oral with metformin^6^.

In all diabetes types, hyperglycemia is induced by a relative or absolute lack of insulin action. Insulin has anti-inflammatory and antioxidant effects such as suppressing proinflammatory transcription factors mediating inflammation and reactive oxygen species (ROS) production^7^. Some studies have reported the autoxidation of glucose and glycosylation in many tissues by the formation of reactive oxygen derivatives in diabetes. Reactive oxygen derivatives are said to be associated with microvascular complications such as eye, kidney and nerve damage and to a lesser extent cardiovascular disease in patients with diabetes^8–11^.

Pteridines, such as neopterin and biopterin, oxidized form of tetrahydrobiopterin (BH4), are involved in many physiological pathways, and these compounds themselves or derivatives can be detected in biological fluids and tissues^6,12^. As it is a sensitive biomarker to detect T helper 1 (Th1) type immune activation in humans, neopterin is a useful biological indicator of inflammatory disease^12^. Neopterin is an oxidized form of 7,8-dihydroneopterin, which is produced upon interferon-gamma (IFN-γ) induction of macrophages. 7,8‐dihydroneopterin is an antioxidant with radical scavenging and chain‐breaking properties. It inhibits peroxyl and hydroxyl radical formation and thiol loss in the organism. Neopterin is a product of 7,8-dihydroneopterin oxidation during the antioxidant reactions^13^. Moreover, BH4 has various roles as cofactor and antioxidant in a wide range of biological processes, including cardiovascular pathologies, monoamine neurotransmitter synthesis, and immune response. These essential functions in different biological processes set the importance of BH4 for homeostasis in the organism^14,15^. Pterins, including BH4, possess antioxidant effects by their ability of scavenging reactive oxygen species^15^.

Tryptophan (Trp) is an essential amino acid for biosynthesis of proteins. Further, Trp is either converted to serotonin by tryptophan-5-hydroxylase enzyme or reduced to kynurenine (Kyn) through kynurenine pathway^12,16^. Induction of IFN-γ by activated T-cells and leukocytes, results in the oxidation of the indole ring with the indolamine-2,3-dioxygenase (IDO) enzyme and the conversion of Trp to Kyn. The enzyme IDO catalyzes the rate-limiting step of IFN-γ induced Trp catabolism^17,18^. Tryptophan deficiency is the basis for the development of depressive disorders, especially in patients with chronic immunopathology^16,19^.

The major aim of this study is to investigate the alteration in tryptophan metabolism, neopterin and biopterin levels in diabetes and compare the results with healthy subjects. Possible changes with single or combined antidiabetic therapy are also evaluated.

## Results

The demographic data of the participants were presented in Table 1. Waist circumference, hemoglobin A1C (HbA1C) and serum glucose levels of the patients were significantly higher than controls (p < 0.01, all). In addition to these parameters, in T2DM and its subgroups, body mass index (BMI) and weight of patients were higher compared to controls (p < 0.01, all). There were no statistical relation between the demographic data and measured parameters among patients except for the positive correlation between blood glucose and HbA1C which showed the history of the diabetic patients glucose status (p < 0.01, R = 0.652).

**Table 1:** Demographic data and characteristics of participants.

Group (n)	Gender (n)		Mean ± SEM							
	Male	Female	Age (years) (min–max)	Height (cm) (min–max)	Weight (kg) (min–max)	Body mass index (kg/m^2^)	Diabetes duration (years)	Waist circumference (cm)	HbA1C (%)	Serum glucose (mg/dL)
Control (30)	20	10	37.0 ± 1.3 (24–51)	166 ± 2.1 (152–183)	70 ± 3.5 (50–110)	25 ± 1	-	82.7 ± 3.6	5.1 ± 0.1	91.6 ± 1.4
**Patients (68)**	30	38	41.6 ± 1.8 (13–83)	166 ± 1.3 (150–193)	76 ± 2.4 (44–137)	28 ± 1	5.6 ± 0.7	97.5 ± 2.2*	8.8 ± 0.3*	178 ± 12*
Diabetes type I (34)	12	22	32.1 ± 2.2 (13–65)	169 ± 1.8 (150–185)	67 ± 2.2 (44–94)	23 ± 1	6.5 ± 1.0	89.2 ± 2.2	9.8 ± 0.4*	223 ± 20*
Diabetes type II (34)	18	16	51.4 ± 1.7* (35–83)	163 ± 1.8 (151–193)	87 ± 3.7* (61–137)	33 ± 2*	4.5 ± 0.8	104.1 ± 2.7*	7.2 ± 0.3*	132 ± 7*
Metformin (16)	10	6	49.7 ± 2.0*^,+^ (35–67)	160 ± 2.0^+^ (151–176)	87.8 ± 6.4*^,+^ (61–132)	34 ± 2*^,+^	3.9 ± 1.2	102.0 ± 4.6*^,+^	6.4 ± 0.2*^,+^	121 ± 12*^,+^
Metformin + Vildagliptin (18)	8	10	52.8 ± 2.7*^,+^ (37–83)	166 ± 2.8 (151–193)	87.2 ± 4.4*^,+^ (69–120)	32 ± 2*^,+^	4.8 ± 1.2	105.4 ± 3.4*^,+^	7.7 ± 0.3*^,+^	141 ± 8*^,+^

*p < 0.01, vs control; ^+^p<0.01, vs Type 1 diabetes.

As shown in Table 2, there was no difference between controls and diabetes patients in terms of serum and urinary neopterin and urinary biopterin levels (Table 2). Serum neopterin levels were significantly lower in patients with T1DM patients compared to T2DM patients (p = 0.029). Urinary biopterin levels of T1DM patients were significantly lower of T2DM patients using both metformin and vildagliptin (p = 0.029).

**Table 2:** Neopterin and biopterin levels of the study groups with standard error of mean.

Group	n	Urinary (µmol/mol creatinine)		Serum neopterin
		Neopterin	Biopterin	(nmol/L)
Controls	30	152.48 ± 10.81	94.91 ± 9.88	6.55 ± 0.39
**Diabetes**	68	175.04 ± 13.85	94.93 ± 9.68	6.87 ± 0.39
Type 1 (Insulin)	34	160.52 ± 18.29	78.86 ± 8.40	6.18 ± 0.47
Type 2	34	190.55 ± 20.90	113.17 ± 17.46	7.58 ± 0.61*
Metformin	16	195.65 ± 44.77	97.96 ± 34.73	7.88 ± 1.16
Metformin + Vildagliptin	18	186.95 ± 18.13	123.91 ± 17.54*	7.32 ± 0.58

* p < 0.05, vs type 1 diabetes.

Serum Kyn-Trp concentrations and the Kyn/Trp levels of participants were detected and results were summarized in Table 3. Diabetic patients were grouped according to their conditions and the treatments they were receiving, the comparison of neopterin and biopterin levels among subgroups was shown in Table 2.

**Table 3:** Kynurenine pathway data of the study groups.

Group	n	Kyn pathway variables (mean ± SEM)		
		Trp (μmol/L)	Kyn (μmol/L)	Kyn/Trp (μmol/mmol)
Control	30	67.62 ± 2.99	1.85 ± 0.12	27.17 ± 1.18
**Patients**	68	62.07 ± 1.53	1.63 ± 0.06	26.43 ± 0.96
Type 1 (insulin)	34	61.19 ± 1.98*	1.55 ± 0.08^+^	25.87 ± 1.47
Type 2		62.97 ± 2.36	1.70 ± 0.10	26.99 ± 1.23
Metformin	16	60.90 ± 3.89	1.58 ± 0.16	25.55 ± 1.74
Metformin + Vildagliptin	18	64.81 ± 2.84	1.81 ± 0.12	28.27 ± 1.74

*p = 0.05, vs control group; ^+^p < 0.05, vs control group.

**Figure 1:**
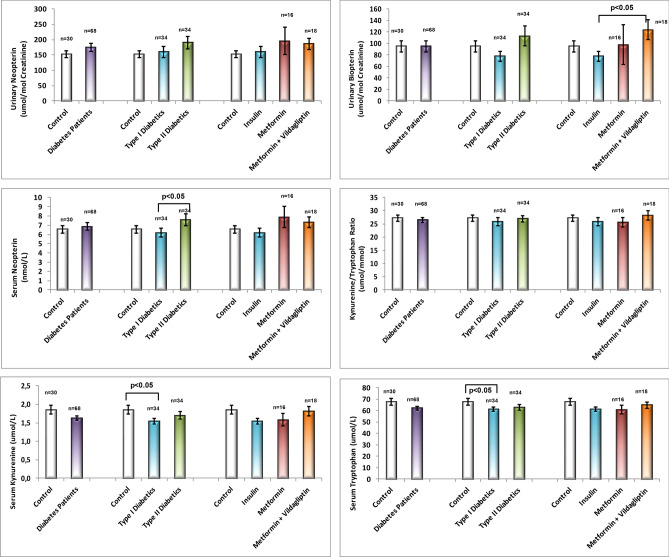
Comparison of the urinary and serum parameters of control and diabetes groups.

Table 3 represents the Trp, Kyn and Kyn/Trp ratios of the study groups. Trp and Kyn levels were significantly different in T1DM patients using insulin compared to the control group (p = 0.05 and p < 0.05, respectively). On the other hand, there was no statistical difference when the control, diabetics and subgroups were compared (p > 0.05, all). Mean values of measured parameters given in Tables 2 and 3 were presented as bar graphs in Fig. 1.

**Figure 2:**
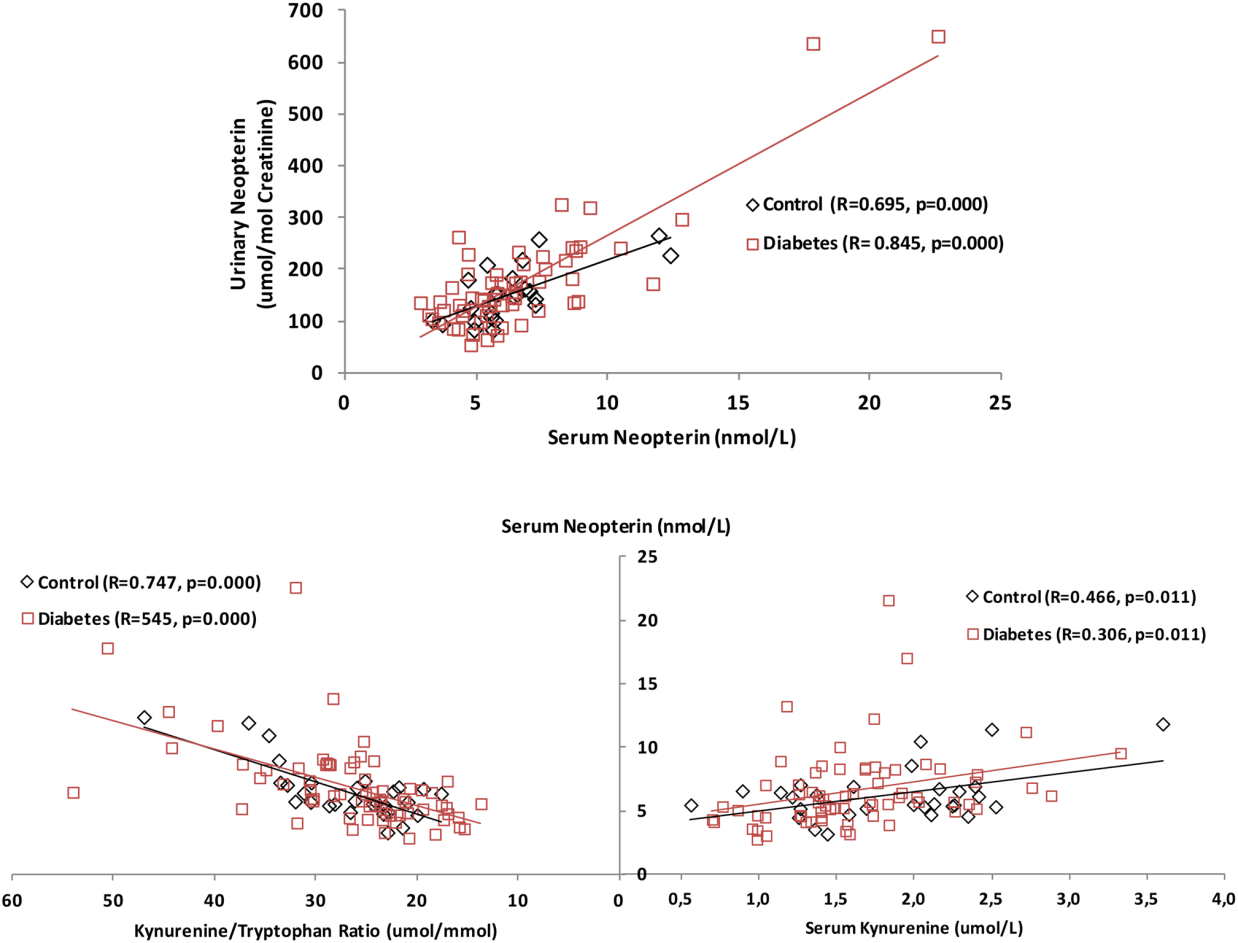
Significant correlations of measured parameters in study groups.

In this study, it was determined that the pathway components of neopterin, biopterin and Kyn in both control and T1DM and T2DM patients did not vary with age of the study participants (p > 0.05, data not shown). However, it was found that the relationship between serum neopterin and urinary neopterin, kynurenine and kynurenine/tryptophan ratio were statistically significant in both control and patient groups as shown in Fig. 2.

## Discussion

Diabetes is an endocrinological disease with high incidence and important complications. The increasing number of diabetic patients notes the importance of current noninvasive biomarkers for early diagnosis. It is reported that the effect of age varies according to the type of diabetes; T1DM is more frequently observed before the age of 25 years^1^, whereas the incidence of the disease increases with age in T2DM^1,2^. In the present study, the mean age of T2DM patients was significantly higher than T1DM patients as expected (p < 0.05). However, when the relationship between diabetes and age is examined, the age did not have any effect on measured parameters in study participants including diabetic subgroups. The pathway components of neopterin, biopterin and Kyn did not change with age in any of the study groups (all, p > 0.05, data not shown). Similar results have also been reported by Dominguez-Rodriguez et al.^24^. In this study, the effect of gender on the measured parameters was also investigated and compatible with previous studies in adults^25,26^, it was found that gender had no effect on measured parameters in neither control nor patient group(s) (all, p > 0.05; data not shown).

In some pathologies, macrophages generate ROS and consequently oxidative stress in addition to neopterin and 7,8-dihydroneopterin when they are stimulated by IFN-γ. Therefore, increased neopterin levels are often detected in oxidative stress related pathologies, and it is reported that neopterin may be an indirect indicator of oxidative stress induced by the immune system^27,28^. Neopterin can be easily measured in biological fluids, and positive strong correlation between serum and urinary neopterin levels show prevalent T-cell mediated immune activation. It has been reported that neopterin levels are higher in diabetes patients^29,30^ and patients with diabetic complications^31–34^. Patients newly diagnosed with T1DM, which is considered as an insulin-dependent chronic autoimmune disease, have higher neopterin concentrations than healthy individuals and that neopterin levels are similar in T2DM to the control group^35^. Similarly, higher serum neopterin levels have been reported in patients with gestational diabetes compared to healthy pregnant women^36,37^. In here, neopterin concentrations in serum and urine samples were positively correlated in both controls and diabetics (R = 0.695, p < 0.01, and R = 0.845, p < 0.01). However; no statistically significant difference was observed between controls and diabetic patients in neopterin levels as shown in Table 2. When serum and urinary neopterin levels of healthy controls and diabetes patients were compared, it was found that these levels were higher in diabetic patients, but this difference was not statistically significant (both, p > 0.05). T2DM patients using oral antidiabetic agents (both single and combination therapy) presented higher serum neopterin levels, that might indicate the systemic inflammation, than T1DM patients having insulin therapy. It has been thought that metformin or metformin + vildagliptin therapy led to inflammation or insulin therapy resulted in an antiinflammatory effect in such a way that T1DM patients presented lower neopterin levels compared to control lacking statistical significance. Nevertheless, similar lower neopterin levels observed in T1DM patients’ urinary neopterin results compared to oral antidiabetic therapy patients (total T2DM) showed no statistical significance. In T2DM, Th1 cells tend to increase in peripheral blood and adipose tissue of patients^38–40^. Sireesh et al.^41^ reported that IFN-γ and related cytokine levels were increased while Th2 response cytokines, having homeostatic effects on oxidative stress and antioxidant status were decreased in T2DM patients. Metformin and DPP4 inhibitors such as vildagliptin have immunomodulatory effects, and they are both reported to decrease IFN-γ production, modulate T lymphocyte activation and decrease Th1 type immune response by diverse mechanisms^42–46^. Serum and urinary neopterin levels in T2DM patients were found higher than T1DM patients (p = 0.029 and p = 0.158, respectively; Table 2). It may be concluded that either oral antidiabetics or insulin has altered T cell activation.

Biopterin, same as neopterin, can be easily detected in urine; biopterin is the final metabolite showing the effects of ROS in pteridine pathway. In this study, there was not any statistical difference between controls and total diabetic patients; nevertheless, patient groups receiving oral diabetic therapy have higher urinary biopterin concentrations than patients on insulin therapy. T1DM patients presented lower biopterin results than patients with oral antidiabetic therapy and even control group. This decrease in urinary biopterin levels reminded of insulin’s antiinflammatory and antioxidative effects. It was also thought that oral antidiabetics increased the excretion of biopterin by affecting the nitric oxide-associated BH4 pathway, which is an indicator of oxidative stress.

In macrophages, induction of IDO is associated with induction of GTP cyclohydroxylase, the key enzyme in pteridine biosynthesis. This parallel induction suggests that synthesis of neopterin and BH4 is also increased. While neopterin production increases in a variety of diseases, slight or no change of biopterin is observed. IFN-γ induces the GTP cyclohydroxylase enzyme which initiates neopterin and BH4 synthesis. On the other hand, it has no effect on the enzymes in BH4 biosynthesis pathway. In this study, the levels of urinary biopterin which is a very important cofactor in the hydroxylation reaction of Trp were measured and BH4 pathway was evaluated indirectly by biopterin excretion. Biopterin levels were not different in diabetes patients compared to control group. However, in patients with T2DM using metformin and vildagliptin, the level of biopterin was found to be 1.5 times higher than that of insulin-controlled T1DM patients. In the subgroup of T2DM using metformin alone, it was found that the level of biopterin was higher than the insulin group, although not significant.

Tryptophan metabolism is triggered by inflammation and/or degeneration in diabetes, causing increased Kyn metabolites^47^. Oxenkurg^25,26^ has reported that, a high level of Kyn/Trp is associated with altered Trp levels in hemodialysis patients with diabetes. In addition, a statistically significant relationship between neopterin levels and the elapsed time after individuals were diagnosed with diabetes was reported^48^. In this study, the possible correlations between measured parameters and the duration of diagnosis were evaluated, but no relationship was found (all, p > 0.05; data not shown). When the levels of Trp, Kyn and Kyn/Trp ratio were evaluated, there was not any statistical difference between controls and total diabetics in Kyn pathway parameters. On the other hand, a statistically significant difference was found between T1DM subgroup patients and controls in Trp and Kyn levels (both, p < 0.05). Besides, it was found that serum neopterin levels and Kyn/Trp ratio were significantly correlated in both controls and total diabetic patients (R = 0.747, p < 0.01 and R = 0.545, p < 0.01). IDO, the rate limiting enzyme of Kyn pathway, is mainly induced by various inflammatory stimuli such as IFN-γ, tumor necrosis factor alpha (TNF-α), interleukin-1 (IL-1), IL-2, tumor growth factor-beta (TGF-β), IL-10, and adenosine^47^. This situation is similar to other studies in which neopterin showing immune activation and Kyn/Trp ratios expressing IDO activity are parallel^49–51^. Inflammatory stimuli via molecules such as IFN-γ triggers both Kyn pathway and T-cell mediated immune response.

In summary, the relationship between elevated neopterin concentrations and increased Trp degradation has been shown in infectious diseases such as HIV, gynecological cancers, malignant tumors, cardiovascular diseases, neurodegenerative diseases, or normal aging process diseases^27,52^. Increased Kyn/Trp ratio in diabetes shows that IDO is activated; this ratio is also associated with neopterin, an indicator of immune activation, pointing out that inflammation and/or immune mediators play role in diabetes. Although some immune markers in the immune system pathway may change as a result of exposure to various xenobiotics, including drugs used in treatment as well as diseases, the use of metformin and/or vildagliptin in T2DM patients did not have any effect on the measured parameters (p > 0.05, all). The essential limitations of this study are the few participants in subgroups and the lack of evaluation of oxidative stress and inflammatory markers such as inflammatory cytokines parallel to the pteridine pathway variables. Further studies with increased number of patients with antidiabetic treatment and evaluating additional parameters may lead us to more precise conclusions.

## Materials and methods

### Study design, participants and sampling

Before the organization of the study groups, sample size was calculated with power analysis, and it was estimated as 25 participants with 95% confidence level and 5% acceptable difference. Afterwards, the study was conducted with 30 healthy controls (37.0 ± 1.3 years) and 68 diabetes patients (41.6 ± 1.8 years). Diabetes patients (38 females and 30 males) were chosen from the volunteers who admitted to the Endocrinology outpatient clinic of the Eskişehir City Hospital during their scheduled control. The comorbities and family histories of the patients were questioned. Of 68 diabetes patients 10 had hypertension, 3 had hyperlipidemia, 7 had thyroid disorder and 1 had neuropathy. Control group (10 females and 20 males) consisted of healthy subjects without any chronic conditions and receiving any medications. All subjects in the study were recruited based on the medical history and clinical examination, and the ongoing therapy protocol of the diabetes patients was not changed. The subgroups of diabetes patients were as follows: 34 patients with T1DM, 16 patients with T2DM metformin treatment, and 18 patients with T2DM metformin and vildagliptin combination treatment which was prescribed for the compliance of patients. Transplant recipients or donors, patients with end-stage renal damage and hemodialysis, acute infection or autoimmune diseases, cancer patients, patients receiving chemotherapy or radiotherapy were not included in the study. Samples were collected in the morning to eliminate circadian rhythm changes. Fasting blood samples were drawn into vaccutainer tubes and centrifuged at 3500 rpm at 4 °C for 10 min to separate the serum fractions. Serum and urine samples were prevented from UV exposure and kept at -80 °C until analysis.

### Measurement

Neopterin, biopterin and creatinine concentrations in urine samples were measured simultaneously by high-performance liquid chromatography (HPLC, HP Agilent 1100, Vienna, Austria). Reversed-phase chromatography was applied with 15 mM phosphate buffer (pH 7) containing 2.5% (v/v) methanol as the mobile phase at a flow rate of 1 ml per minute. Neopterin and biopterin levels were measured with fluorescence detector at 353 nm excitation and at 438 nm emission wavelengths, creatinine levels were simultaneously measured at 235 nm with UV detector (HP Agilent 1100, Vienna, Austria). Urinary neopterin and biopterin levels were expressed as μmol/mol creatinine^20,21^. The coefficient of variance was calculated as 3.9% for within-run and 2.7% for between-run analysis.

Serum neopterin concentrations were analyzed with commercial enzyme-linked immunosorbent assay (ELISA) kits (IBL, Hamburg, Germany) according to manufacturer’s instructions. Optical density was measured at 450 nm with microplate reader (Spectra Max M2, England). Serum neopterin levels were expressed as nmol/L.

Serum Trp and Kyn concentrations were determined by HPLC (HP Agilent 1100, Vienna, Austria). Mobile phase was consisted of 15 mM pH 6.4 KH_2_PO_4_ buffer containing 0.7% acetonitrile at a flow rate of 0.8 ml per minute. Trp was detected with a fluorescence detector (excitation 286 nm, emission 366 nm). Kyn was detected simultaneously with a UV detector at 360 nm. Kyn to Trp ratio was expressed in μmol/mmol^22,23^. The coefficient of variance for Kyn/Trp was calculated as 3.60% for within-run and 5.72%for between-run analysis.

### Statistics

Statistical Package for Social Sciences (SPSS) 11.5 was used to analyze the data. Spearman correlation analysis and simple regression analysis were used to evaluate the relationships between descriptive statistics and dependent variables. Kruskal–Wallis analysis was used to evaluate the differences between the groups and Mann–Whitney *U* test was used to evaluate the independent subgroups. The results are presented as arithmetic mean ± standard error. *P-*values less than 0.05 were considered to indicate statistical significance.

### Ethical approval

All the methods in the study were approved by the Ethical Committee of Scientific Research and Clinical Trials of the Turkish Ministry of Health Eskişehir City Hospital (Date: 14/05/2016, #22205031–060.99/12). The study was carried out in accordance with the statement of Helsinki Declaration. Participants were informed about all aspects of the study and written informed consent was obtained from all participants themselves.
